# Perceived quality of life among Visceral Leishmaniasis and HIV coinfected migrant male-workers in Northwest Ethiopia: a qualitative study

**DOI:** 10.1186/s12889-017-4132-z

**Published:** 2017-02-16

**Authors:** Mekuriaw Alemayehu, Mamo Wubshet, Nebiyu Mesfin, Abebaw Gebayehu

**Affiliations:** 1 Institute of Public Health, College of Medicine and Health Sciences, University of Gondar, Northwest Ethiopia, P. O. Box - 196, Gondar, Ethiopia; 2 Department of Public Health, St. Paul’s Hospital Millennium Medical College, Addis Ababa, Ethiopia; 3 School of Medicine, College of Medicine and Health Sciences, University of Gondar, P. O. Box - 196, Gondar, Ethiopia

**Keywords:** Quality of life, Qualitative study, Visceral Leishmaniasis, HIV coinfection

## Abstract

**Background:**

There is paucity of data on quality of life as a dimension of treatment outcome among Visceral Leishmaniasis and HIV coinfected patients. This study sought to explore perceived quality of life among Visceral Leishmaniasis and HIV coinfected male migrant workers in Northwest Ethiopia.

**Methods:**

Twenty Visceral Leishmaniasis and HIV coinfected study participants took part in the in-depth interviews at Visceral Leishmaniasis and HIV treatment centers. Ten participants were on antiretroviral treatment (ART) and the remaining 10 have not yet started ART. All interviews were recorded, transcribed and translated for analysis. Data were analyzed by qualitative content analysis using Open Code software version 3.4.

**Result:**

Participants reported on four aspects of quality of life: liveability of the environment, utility of life, life ability of a person and appreciation of life. Respondents living environment, therapeutic side effects of Visceral Leishmaniasis drugs, poverty and stigma negatively affected their quality of life. On the contrary, good treatment response and financial security were reported to positively affect their quality of life.

**Conclusion:**

Challenges related to the living environment, financial limitations and sub-optimal response of Visceral Leishmaniasis drug and relapse of Visceral Leishmaniasis disease are factors most negatively affecting the quality of life of Visceral Leishmaniasis and HIV coinfected patients. Micro-financing and other socio-economical support programs should be launched to assist the unemployed males migrating to Visceral Leishmaniasis endemic and relatively higher HIV prevalent areas to work as daily laborers. HIV prevention programs in HIV positive-living counseling programs should target such high risk migrant workers in the endemic areas.

## Background

Human immunodeficiency virus (HIV) and Visceral Leishmaniasis (VL; also known as “Kala-azar”) coinfection reported in 35 countries [1, 2], among which Ethiopia carries the highest burden. Twenty three percent (23%) of people with VL were also infected with HIV in 2008, far higher than anywhere else in the world. The affected populations are largely very poor male seasonal migrant workers that travel in the harvesting season from non endemic highlands to the cotton, sesame and sorghum fields of Humara and Metama on the Sudanese boarders [3, 4].

VL and HIV coinfection are associated with special therapeutic challenges. VL-HIV coinfected patients are at higher risk of relapse and death. Furthermore, VL adversely affects the response to antiretroviral therapy (ART) [5, 6]. In Ethiopia, free ART has been given to patients since 2003 [7]. To improve quality of life (QoL), it is vital to explore the perception and determinants of QoL.

Many studies have provided important information on the correlates of health related quality of life (HRQoL) during HIV infection. Several studies have documented significant improvements in QoL during ART [8–10]. There is however knowledge gap on the understanding of perception of QoL of patients with VL-HIV coinfection as a treatment outcome. Thus, the aim of this study was to explore perceived QoL among VL-HIV coinfected migrant male-workers using qualitative method, and to reveal the concept of QoL for people who are VL-HIV coinfected in Ethiopian context to contribute to the development of VL-HIV coinfected persons care model.

## Methods

### Study design

An in-depth qualitative, phenomenological study design was selected to carry out searching of the lived experience and perceptions of the study subjects on the quality of life of VL-HIV coinfected male patients that were receiving VL treatment.

### Study setting

The study was carried out at three different sites found in Northwest Ethiopia. The first site was Abdrafi inpatient kala-azar treatment center located in Abdrafi; at this health center medical services are provided for patients with Leishmaniasis, VL-HIV coinfection and snake bite. The second site was Kala-azar Treatment and Research Center found in the University of Gondar Hospital located in Gondar; at this center both outpatient and inpatient medical services are provided for patients with Leishmaniasis and VL-HIV coinfection in addition to the comprehensive medical service from other units in the University of Gondar Hospital. The third site was Kahsay Aberra Hospital located in Humera kala-azar treatment center; at this center both outpatient and inpatient medical services are provided for patients with Leishmaniasis, VL-HIV coinfection and many other hospital level services. Study participants come from different parts of kala-azar endemic areas with a variety of cultural background in Northwest Ethiopia. As the typical patient approach procedure, when the patients come to the clinic, the physicians and the health officers will do clinical and laboratory examinations to diagnose the patients for kala-azar, if patients are found to be positive for VL they will further screen them for HIV. This allows the health workers to select the appropriate treatment for VL-HIV coinfection.

### Recruitment of participants

The study included twenty VL-HIV coinfected male patients who were attending VL treatment. Newly diagnosed VL-HIV coinfected patients who already started ART and who didn’t start ART were included in this study. We distributed invitation letters to approach the study participants at kala-azar treatment centers of the selected health facilities in the study area. A purposive sample of 20 study participants, ten patients who already started ART and the remaining ten patients didn’t start ART, participated in the study. Study participants had not been evaluated for quality of life before this study. Individuals who were seriously sick and mentally disabled were excluded.

### Data collection and analysis

The data collection method was in-depth semi-structured interviews. After securing written informed consent, a topic guide was used as a prompt for questioning. The key consideration for influencing the direction of each interview was the participant’s response to questions in terms of their individual experiences. The principal investigator (PI) conducted the face-to-face in-depth interviews using semi-structured guide in venues suitable for the participants. The guide was developed in English and translated in to Amharic (the local language) for all participants. Interviews were recorded using audio recorder devise in order to capture all information provided by the interviewee.

The interview was started by couple of questions prepared to create rapport with the study participants. These questions focus on the life experiences as a migrant worker and challenges they faced when they are diagnosed with VL-HIV coinfection. After we created a rapport, the theme of quality of life issues was introduced in subsequent interview of persons living with VL-HIV coinfection. The interviewer probed for any changes in personal values, responses for treatments and goals and how they are related to quality of life.

The PI had continued interviewing participants until no new information is provided from additional interviews (i.e., the point of saturation is reached). Upon completion of each interview and based on the recorded audio the PI developed a detailed transcription that captures all the discussion. These texts and the emerging themes identified during the course of the interviews were confirmed by the coauthors and issues were addressed. All the interviews were transcribed and prepared for coding and thematic analysis. The typed data were analyzed by qualitative content analysis using the Open Code software version 3.4 (Umea University, Umea, Sweden). The Open Code software is used as a tool for classifying and sorting qualitative information.

The investigators scrutinized transcripts were read multiple times in order to get general sense of the content. An inductive qualitative content analysis method was used to analyze the data. A coding framework was developed based on the emerging themes. Emergent categories and themes were identified based on meticulous and systematic reading and coding of the transcripts. Codes and sub-codes were refined during the analysis. This was done by the PI and reviewed by the coauthors.

## Results

Among the twenty patients included in the study, ten patients were started taking antiretroviral (ARV) drugs in addition to the Visceral Leishmaniasis (VL) drugs during interview. The remaining ten patients have not yet started taking ARV drugs but were taking VL treatment. All of the study participants were male migrant workers. The age range of participants was from 25 to 52 years. Emergent categories and themes were identified from the interviews, including “Liveability of the environment” as indicators of QoL; “Utility of life”, covers responses of life expectations/goals; “Life ability”, covering responses to VL and HIV treatments, stigma, stress and VL relapse and appreciation of life.

### Liveability of the environment

#### Indicators of quality of life

Quality of life was discussed in terms of wellbeing and happiness influenced by good health, having money, good living environment, good social relations and emotional wellbeing. All of the study participants (taking ART and not taking ART) interviewed defined QoL in a way similar to World Health Organization (WHO) definition of health as “a state of complete physical, mental and social wellbeing and not merely absence of disease or infirmity” [11].

One 51 year-old man, taking both VL and ARV drugs said “QoL is about being healthy, having good energy and enough money.” A 42 year-old man, taking both VL and ARV drugs remarked “QoL is when one has health, access to VL treatment centers and money. If I have money and access to VL treatment center, I am able to treat my VL relapse immediately; my QoL is good”. A 39 year-old man, taking only VL drug and not in ART said “QoL is about not working in hot areas (low lands) and having work in cold areas (high lands), having shelter”. A 33 year-old man, taking both VL and ARV drugs said “QoL is about having money, not doing laborious work, free from any substance use or abuse such as chewing khat, smoking cigarette and drinking alcohol and not being coinfected by VL”. Another 29 year-old man, taking only VL drug and not in ART said “It’s about being cured from VL, able to create my own business and earn money and marrying a woman and have children”.

The majority (16/20; 80%) of the study participants in both groups mentioned availability of money is very much vital for their wellbeing and hence a good QoL, and for example “for me after I finished VL treatment, I don’t have money for transport to go back to my residence. To cover my daily expenses I need to have money” (34 year-old man, taking only VL drug and not in ART) and “I need money to pay my house rent, for food and to buy clothes that are vital for sustaining my life” (42 year-old, taking both VL and ARV drugs). One 32 year-old man, taking both VL and ARV drugs said that “I am very much worried about my future life after I am discharged from this VL treatment center. Unlike previous times I don’t have enough energy to work as a daily laborer in hot areas (low lands) because VL infection severely weakened my body. On the other hand, in order to do my own job, I don’t have enough money. I am very much worried about how to get money for my daily subsistence. It is very much difficult to be happy and lead good QoL when you have no money”.

All of the respondents in both groups stated that good health and money were crucial to their wellbeing and QoL. Most of the participants were migrant daily laborers. They were more frank when discussing money as a source of wellbeing. For instance, one 34 year-old migrant daily laborer resided in May-kadra district who are taking only VL drug and not in ART said that “my choice of living in May-kadra is because there is job opportunity and I am able to earn money” and also one 33 year-old migrant daily laborer residing in Humera district who are taking both VL and ARV drugs stated “In order to sustain my income, I am working laborious work in very hot area of Humera districts”.

Almost all (19/20; 95%) interviewees in both groups highlighted living environment as a cornerstone of their not wellbeing and QoL, for example “I have resided and spent many years in very hot area because I can be easily employed as a daily laborer in large farms and I can generate money easily. It is not suitable for health because there are diseases like malaria and VL” (42 year-old man, taking both VL and ARV drugs) and “I am living in a hot and VL disease prevalent area. For this reason I am not happy in my living environment and withstanding all those problems I will continue working as a daily laborer to sustain my income” (33 year-old man, taking both VL and ARV drugs). One 29 year-old man taking only VL drugs and not in ART said “I am working as a daily laborer moving from one hot area to the other hot area in order to generate money for my daily expenses. Those areas I have visited were very hot and were not suitable for health because there are different diseases like malaria, VL and diarrheal diseases”.

Lack of happiness due to living environment was reported by majority (18/20; 90%) of the respondents in both groups. For instance, one 39 year-old man, taking only VL drugs and not in ART said that he is fade up of working as daily laborer in “berha”, translated as “very hot areas or low lands”. When asked what exactly he meant by this, he explained that these areas are extremely hot and there are different diseases like VL. Therefore, working in those areas were made me coinfected with VL and it makes me very much weak.

Social relationships including support from immediate family reported by few (2 patients or 10%) of the respondents in both groups as a key to their wellbeing, for example, “I have supportive family, my mother’s wellbeing and successful friends around me makes me happy” (52 year-old man, taking both VL and ARV drugs) and “a good relationship with my wife and the care I get from my brother makes me happy” (45 year-old man, taking only VL drugs and not in ART).

Emotional stability was a concern among a few (1 patient or 5%) of those interviewed. A 45 year-old man, taking only VL drugs and not in ART described QoL as “Living the life without worries is having good QoL”. As happiness was stated by all of the participants, we asked them further what was meant by happiness for VL-HIV coinfected patient. Happiness was seen as indicator of QoL. The main factors remarked as a source of happiness were to get cured from VL disease and the free treatment they got from the treatment facilities. The other factors mentioned as a source of happiness included financial wellbeing. These findings were consistent across all ages and for both groups (with and without ART) of the participant.

Almost all (19/20; 95%) of the study participants in both groups clearly mentioned that recovering from VL disease and the treatment centers where they have received care were the source of their happiness. One 25 year-old man taking only VL drugs and not in ART said “my source of happiness depends on being cured from VL disease”. Another 34 year-old man, taking only VL drugs and not in ART stated that “the only way to get back my happiness depends on being cured from VL disease”. A 33 year-old man, taking both VL and ARV drugs remarked “my happiness entirely depends on recovering from VL disease. I am also very much happy by free treatment that I got from this treatment center”. One 41 year-old man, taking both VL and ARV drugs said that “My source of happiness entirely depends on the fact that I am cured from VL disease and the support I got from the treatment center. In the last 5 years I had experienced eleven times VL relapse and in all those relapses this treatment center was giving me free treatment”.

All (20 patients or 100%) of the participants in both groups were mentioned lack of economical wellbeing as a major contributor to their unhappiness. According to one 34 year-old man, taking only VL drugs and not in ART said “In order to lead my life properly I need money and money is everything. I don’t have money to cover my daily expenses. If I don’t get money to cover the cost of a transport from the treatment center to my home town then I will be a beggar”. A 42 year-old man, taking both VL and ARV drugs said “money is a source of my happiness. For instance, if I didn’t have money I wouldn’t reach here to follow my treatment. Hence, when I have money in my pocket I do have a better happiness. Currently I don’t have money and I feel very much angry”. A 51 year-old man, taking both VL and ARV drugs said “I feel very much angry when I have no money; I am not a happy person because I don’t have money to pay for house rent and to buy my daily food consumption”.

### Life ability

#### Response to VL and ARV drug treatment

Majority (14/20; 70%) of the participants in both groups were given Liposomal amphotricine B (AmBisome, Gilead Sciences Ltd, Paris, France) and some (6/20; 30%) received a combination of AmBisome with oral Miltefosine for VL treatment. Most (10/14; 71%) of the participants in both groups who received only AmBisome reported side effects. Those who had side effects said that they cleared within a couple of days and that they felt much better. A 51 year-old man, taking both AmBisome and ARV drugs said “the first few days may be four to five days were bad, I was unconscious for the first day, the next 3 to 4 days I had experienced severe sweating, abdominal cramp, constipation, sense of burns around injection areas and series of vomiting and later stabilized”. Another 25 year-old man, taking only AmBisome and not in ART also stated “the first couple of days were bad, I experienced series of vomiting that included blood, lower back pain, variability of body temperature and loss of appetite and then after few days these were ceased”.

Two third (4/6 or 2/3rd; 67%) of the participants in both groups who received a combination of treatment were also reported side effects, for example, “After I have taken VL drugs, I experienced stomachache, constipation, feeling pain during urination, loss of appetite and severe lower back pain” (34 year-old man, taking only combination of AmBisome with oral Miltefosine and not in ART), and “the side effects that I experienced because of VL drugs are diarrheal disease and loss of appetite and whenever I took Miltefosine without eating food I felt stomachache and vomiting” (41 year-old man, taking both combination of AmBisome with oral Miltefosine and ARV drugs).

A 45 year-old man who is in ART and he had been experienced seven times VL relapses in the past four years said “Among all side effects of VL treatments that I experienced during my VL relapse times; my second time VL relapse treatment were very much severe. The drug I had taken during this time was sodium stibogluconate (SSG). After nine days of taking SSG I experienced that around two liters of blood came out from my mouth and nose and later I quit the drug and the physician changed it to AmBisome”.

Some of the participants in ART group reported a reduction in therapeutic response of VL treatment and this results high rate of relapse, for example, “I have experienced six times relapse. In most cases the VL relapse occurs when I am starved and when I am engaged in laborious works in very hot areas (low lands)” (39 year-old man, taking both VL and ARV drugs) and “Usually my VL relapses when I am engaged in laborious work in very hot areas” (33 year-old migrant daily laborer, taking both VL and ARV drugs). A 41 year-old man, taking both VL and ARV drugs remarked “In the last five years I had experienced 11 times VL relapse because of this reason I couldn’t manage to do work and able to generate money for my daily expenses”.

Most (8/10; 80%) of the participants who are in ART group reported minor side effects of ARV drug and these side effects were cleared within few days and then they felt much better. Those who had these side effects of ARV drug perceived the side effects as very minor compared to the benefits of taking ARV: “The first few days may be 2 or 3 days were bad, I felt sleep disorder and dizziness and later stabilized” (27 year-old man, taking both VL and ARV drugs). Another 33 year-old man, taking both VL and ARV drugs said “For the eight days since I started ARV, I experienced bad dreams and sleep disorder then after eight days these feelings were ceased”. One 29 year-old man, taking both VL and ARV drugs stated “The first eight days were bad; I had sleep disturbance and terrible dreams which later ceased. Sometimes I felt headache after I have taken ARV drug”.

Almost all (9/10; 90%) of participants who were not in ART have high interest to start taking ARV drugs. The reason of not starting ARV drugs was residence of the participant. All (10 patients or 100%) of the participants who have not in ART are migrated from their home town to be employed as daily laborer in study areas. Therefore, after they completed VL treatments they will be linked to ART centers that are found in their permanent residence or home town. A 34 year-old man, taking only VL drugs and not in ART said “After I completed my VL treatment, I will start ARV drug in May-kadra health center which is found in my home town”. Another 36 year-old man, taking only VL drugs and not in ART stated “Till now I didn’t start ARV drug. The reason for this is I planned to start the drug in my home town ART center”. One 42 year-old migrant daily laborer, taking only VL drugs and not in ART said “I have an interest to start ARV drug but I fear to communicate my HIV status to my wife. The reason for this is my wife is very rigid and I am in deep fear that she will not accept me. My last option will be requesting divorce without telling my HIV status. If it is successful I will start the drug otherwise I will not start it”.

#### Stigma

The majority (8/10; 80%) of the study participants who are in ART reported stigmatization due to their HIV status. Some (2/10; 20%) respondent’s who are in ART seemed to have convinced themselves about their HIV infection and didn’t worry about what other people said or thought of them. A 42 year-old man, taking both VL and ARV drugs said “In my village there is a trend of disgracing a person when he is HIV infected. Because of this reason no one knows about my HIV status and I will keep it secret from my family, friends and the community at large”. Another 39 year-old man, taking both VL and ARV drugs stated “After I told my HIV status to my best friend he refused to eat with me”. Similarly, one 32 year-old man, taking both VL and ARV drugs reported that “After I disclose my HIV status to my best friend and his wife, she refused to eat with me”.

Many of the respondents were migrant daily laborers and they are supposed to work in group in big farm lands – thus making clear their HIV status makes them discriminated by the group. One 42 year-old man, taking both VL and ARV drugs said “I used to work in a group as a daily laborer in Abdrafi farm lands. Among the group one of my friends knew about my HIV status. One day in the middle of the day I felt tired and decided to have nap. During this time my friend told the group about my HIV status and then the group refused to eat and work together with me”.

A few (2/10; 20%) respondent’s who are in ART reported no sense of stigma. One 45 year-old man, taking both VL and ARV drugs said “some people gave me an advice not to take ARV drugs in front of the people but I don’t mind if people know. I always share my experience and how good my life is with the HIV treatment. I encourage people whom I knew to know their HIV status and to act accordingly”. A 41 year-old man, taking both VL and ARV drugs remarked “I don’t care if people know my HIV status. I don’t bother about what other people say about me”.

#### Stress/depression/anxiety/fear

All (20 patients or 100%) of the participants in both group interviewed reported that they were fearful, shocked and worried at the time of VL and HIV concurrent diagnosis. Though, they got support from health care providers. A 34 year-old man, taking only VL drugs and not in ART described his reaction after he was tested VL and HIV coinfection “I was very much depressed and stressed. I thought I was going to die”. Another 41 year-old man, taking both VL and ARV drugs who had been experienced eleven times relapse said “I am depressed and worried about my future life. The multiple VL relapses that I have experienced make me desperate. Many times I felt angry and stressed”. One 25 year-old man, taking only VL drugs and not in ART said “The first couple of weeks were bad, I felt stress and fear to die and later I stabilized quickly after VL and HIV treatment was started”. Participants expressed anxiety about house rent and daily subsistence “Currently I have no money and enough energy to work as a daily laborer; these are my major problems,” remarked a 51 year-old man, taking both VL and ARV drugs.

### Utility of life

#### Goals and expectations

Over a half (12 patients or 60%) of the participants in both groups were hopeful and have set goals for their future life. There were similarities in the desire expressed by the majority of hopeful respondents regarding their wish to gain enough money for creating their own job. The participants’ future plans included relocating their living environment to cold areas, opening mini shop to sustain their income, building their own homes, getting married for those that were unmarried and having children. Plans were explained:

“I hope to make more money and opens my own hotel” (25 year-old man, taking only VL drugs and not in ART), “I would like to relocate from very hot area to cold area like Gondar town; I would also like to be a merchant and get married to have children” (33 year-old man, taking both VL and ARV drugs), “I would like to make more money and relocate to cold areas; I would also open mini shop to sustain my income and I will marry a woman of the same status” (42 year-old man, taking both VL and ARV drugs), “I would like to change my profession, build home in the outskirt of the town, and buy cattle, sheep and hens so that I will get non-laborious work” (45 year-old man, taking both VL and ARV drugs).

A few (2/10; 20%) interviewees who are in ART have changed habits such as chewing khat and drinking alcohols because of frequent relapses of VL, and put increased focus on planning for their future how not to be coinfected with VL again. “My VL relapses were occurred when I consecutively engaged in substance use/abuse like khat chewing and drinking alcohol. Therefore, in order to prevent VL relapse I have stopped khat chewing and drinking alcohol” (39 year-old man who was taking both VL and ARV drugs and experienced six times relapse), “I used to drink alcohol and smoke cigarette. I stopped these activities since I started treatment. I live my life free from any substance use/abuse to prevent VL relapse” (40 year-old man, taking both VL and ARV drugs).

Some (8/20; 40%) of the study participants in both groups were not hopeful about their future, for example, “My future is dark because I don’t have enough energy to work as a daily laborer. I am also desperate for not establishing family” (34 year-old man, taking only VL drugs and not in ART), “I don’t have future because if I wasn’t infected with HIV, I would have married and have children” (45 year-old man, taking only VL drugs and not in ART). One 41 year-old man who was taking both VL and ARV drugs and experienced eleven times VL relapse said “There is no future for me because the frequent relapse of VL makes me very much week and I have no money to lead my life and to establish family”.

### Appreciation of life

QoL by VL-HIV coinfected patients was commonly explained by both groups as being cured from VL disease, change living environment from very hot areas to cold area town and opening mini shop to sustain their income. One 34 year-old man, taking only VL drugs and not in ART said “when coinfected patient cured from VL disease, they have no problems and whatever they set out to do comes out as desired like building home and opening mini shop to sustain their income”. One 51 year-old man who was taking both VL and ARV drugs and experienced six times relapse explain “It’s when one prevents VL relapse, suffers no illness and has mini shop or micro business to earn money for their daily subsistence like eat, drink, and no worries”. Another 33 year-old man, taking both VL and ARV drugs said “It’s about curing from VL disease, when you are healthy and living in cold areas. In addition, when you have income generating means like mini shop for making your basic needs satisfied”.

## Discussion

This study showed that the VL-HIV coinfected patients have to overcome poor QoL; and, special emphasis should be given for the complex challenges existing in the treatment and care of VL-HIV coinfected patients. The findings of this research article were framed in accordance with the Ruut Veenhoven model of “four qualities of life” [12]. Participants’ narratives were alluded to the concept of “liveability of the environment”, which could be considered to explain the prevailing economic status such as a lack of money, living environment and the nature of their work. Moreover, “Life ability of the person” could be defined as how capable one is able to cope with life problems. The participants in our study discussed the side effects of VL and HIV treatments, feelings of stigma, and stress/depression/anxiety/fear. Some of the participants report the ineffectiveness of VL treatments and this results a high rate of relapse. The third theme raised was “utility of life”, which could be considered to describe their expectations/goals of their future life. Some of the patients remarked that relocating from their residence to cold areas and changing job are good for improvement of QoL. Some of the patients also said that the future is very dark. Finally, study participants spoke of subjective wellbeing, expressed as “life in the eyes of beholder” [12]. Majority of the patients to appreciate and enjoy life we should be cured from VL disease, change living environment and opening mini shop. Overall, QoL was perceived in terms of cured from VL disease and opening mini shop.

Living environment was mentioned as indicator of poor QoL by our study participants. Living in very hot lowland areas was a source of VL infection and hence results a poor QoL, all of the participants reporting that their VL infection or relapse were because of their living environment. Our finding was supported by the eco-epidemiology study of VL in Ethiopia [13], the Humera plains in the Tigray and Amhara regional states, bordering Sudan and Eritrea, constitute the main VL endemic area in the country, contributing over 60% to the burden of VL infection. Hence, the finding of this study also gives further evidence on the high transmission of VL from which the clinical cases are reported in Northwest Ethiopia.

The study participants in this study thoroughly discussed their quality of life. They have mentioned the most important aspects in their lives and that have been related to their experience of living with VL-HIV coinfection. The ability to work and earn money to sustain their daily subsistence was described as a major component of their quality of life. Qualitative studies on wellbeing of HIV infected patients in some of the developing countries including Ethiopia, mentioned similar theme and determinants of wellbeing found in our study group. Food, money, shelter, health, family relations, children and education were commonly considered important for wellbeing in population groups [14, 15].

The availability of VL treatment has had a significant impact on QoL of coinfected patients. Most of the patients who received AmBisome and combination with oral Milefosine for VL treatment reported slight side effects. Those who had experienced side effects said that they got relief within a couple of days and that they felt much better. Some of the participants who had a history of VL reported that a reduction therapeutic response of VL treatment and this results high rates of relapses. These findings were supported by other quantitative studies that are conducted on long term effectiveness and treatment outcomes of VL-HIV coinfected patients treated with AmBisome [16]. The VL-HIV coinfected patients showed initial cure following treatment with AmBisome but these patients appear to have much higher rates of VL relapse and mortality than patients not known to be HIV-positive.

Access to the VL-HIV treatment centers has had a contribution to the positive changes in livelihoods of some VL-HIV coinfected patients and it results hopefulness for their future. There were similarities in the desire expressed by the majority of hopeful respondents to gain money for opening mini shop to sustain their income and changing their living environment to non VL endemic areas. This finding is supported by a study from South Africa that elaborates in further details on how HIV patients regained health and social relations while in care [17]. Whereas some of the participants in our study were not hopeful about their future, this finding also in line with the study participants in rural Uganda struggled to “get back to normal” and rebuild their social life’s after diagnosis of HIV and treatment [18]. The present study took place in VL-HIV coinfection treatment facilities. All of the patients offered free treatments for both VL and HIV infections. Half of the included patients were newly diagnosed VL-HIV coinfected patients. Indeed, some even reported a frequent relapse of VL after they are attending the treatment center. Therefore, life disruption was experienced by some of our study participants.

Majority of the study participants’ who were in ART demonstrate that they continue to quietly suffer from stigmatization, stress, poverty, uncertainty about the future and an inability to make known their HIV status. Participants reported experiences of varying degrees of stigma. These results are similar to those of other studies in both developing countries [15, 19] and developed world [20]. These challenges surrounding social stigma and non-disclosure of HIV status negatively affected respondents’ QoL. However, those study participants who had not been on ART treatment during data collection were not reported about stigmatization. The reason for this is we included newly HIV diagnosed patients and they didn’t have exposure with the community with known HIV status.

The limitation of this study is clearly stated by the following statements. All interviews were conducted by the PI and this may have introduced some bias however tape record were played for the study participants and confirmed by their signature and transcripts were also reviewed by colleagues. The present study participants were only male migrant daily laborers this may also introduce some bias. The reason for not including female sex was the difficulty of finding infected female migrant daily laborers. Interviews were tape recorded and this may also have introduced some bias because some of the interviewees were not comfortable with the audio recorder practice due to stigma and non disclosure of the status.

## Conclusion

This study highlights the complexities and multi-dimensional nature of QoL and provided the challenges and concerns faced by those VL-HIV coinfected male patients in an African setting. Challenges of living environment and therapeutic response of VL treatment are factors that most negatively affect the QoL of VL-HIV coinfected patients. Engaging the unemployed males who are desperately migrating to VL endemic and high HIV prevalent areas for job with micro-finance programs and giving technical support to help them create their own job will not only improve the quality of life of the already VL-HIV coinfected individuals but also will alter the transmission dynamics of both HIV and VL. It is possible to plan control programs specific to the endemic areas if the unpredictable nature of the migration dynamics is not interfering with the policy and programmatic planning and intervention. Clinical trials aiming at reducing side effects and improving efficacy should be encouraged. HIV prevention programs and HIV positive-living counseling programs should target migrant workers to VL endemic areas in the country.

Despite the free treatment of VL-HIV coinfected patients provided by the treatment facilities found in Northwest Ethiopia, those who faced a frequent relapse of VL experienced life disruption. Although some participants spoke hope for the future, there are fundamental factors that cause adverse impact on QoL of VL-HIV coinfected patients. These factors were relapse of VL, stress, stigma, poverty and depression. These factors need to be targeted and addressed when managing these patients.

## Abbreviations

ART: Antiretroviral therapy
ARV: Antiretroviral
HIV: Human immunodeficiency virus
HRQoL: Health related quality of life
IDIs: In depth interviews
PI: Principal Investigator
PLHA: People living with HIV/AIDS
QoL: Quality of life
SSG: Stibogluconate
VL: Visceral Leishmaniasis
WHO: World Health Organization
